# Application of Semi-Mechanistic Pharmacokinetic and Pharmacodynamic Model in Antimicrobial Resistance

**DOI:** 10.3390/pharmaceutics14020246

**Published:** 2022-01-21

**Authors:** Kun Mi, Kaixiang Zhou, Lei Sun, Yixuan Hou, Wenjin Ma, Xiangyue Xu, Meixia Huo, Zhenli Liu, Lingli Huang

**Affiliations:** 1 National Reference Laboratory of Veterinary Drug Residues (HZAU), Wuhan 430000, China; mikun@webmail.hzau.edu.cn (K.M.); sunlei23@webmail.hzau.edu.cn (L.S.); liuzhli009@mail.hzau.edu.cn (Z.L.); 2 MAO Key Laboratory for Detection of Veterinary Drug Residues, Huazhong Agricultural University, Wuhan 430000, China; flyingkai@webmail.hzau.edu.cn (K.Z.); hyx97@webmail.hzau.edu.cn (Y.H.); mawenjin@webmail.hzau.edu.cn (W.M.); HuoMeixia@webmail.hzau.edu.cn (M.H.); 3 MOA Laboratory for Risk Assessment of Quality and Safety of Livestock and Poultry Products, Huazhong Agricultural University, Wuhan 430000, China; xuxiangyue@webmail.hzau.edu.cn

**Keywords:** semi-mechanistic PK/PD model, antimicrobial resistance, mathematical equation, dosage regimen

## Abstract

Antimicrobial resistance is a major public health issue. The pharmacokinetic/pharmacodynamic (PK/PD) model is an essential tool to optimize dosage regimens and alleviate the emergence of resistance. The semi-mechanistic PK/PD model is a mathematical quantitative tool to capture the relationship between dose, exposure, and response, in terms of the mechanism. Understanding the different resistant mechanisms of bacteria to various antibacterials and presenting this as mathematical equations, the semi-mechanistic PK/PD model can capture and simulate the progress of bacterial growth and the variation in susceptibility. In this review, we outline the bacterial growth model and antibacterial effect model, including different resistant mechanisms, such as persisting resistance, adaptive resistance, and pre-existing resistance, of antibacterials against bacteria. The application of the semi-mechanistic PK/PD model, such as the determination of PK/PD breakpoints, combination therapy, and dosage optimization, are also summarized. Additionally, it is important to integrate the PD effect, such as the inoculum effect and host response, in order to develop a comprehensive mechanism model. In conclusion, with the semi-mechanistic PK/PD model, the dosage regimen can be reasonably determined, which can suppress bacterial growth and resistance development.

## 1. Introduction

The emerging antimicrobial resistance (AMR) of bacteria threatens clinical therapeutics. The selective pressure exercised by the misuse and abuse of antimicrobials is an important consideration for the antimicrobial industry. In addition, the use of antimicrobials in animals has been linked to a rise in AMR infections in both animals and humans. AMR significantly increases morbidity, mortality, hospital length of stay, direct healthcare-related costs, and the indirect societal costs of infections [1]. There is also a higher excess expenditure of $20 billion in annual health care costs and $35 billion in societal costs due to AMR [2]. Without the effective prevention and control of AMR, it is assumed that AMR-related deaths will increase from 700,000 to 10 million by the 2050s; thus, it is essential to limit the emergence and spread of AMR.

**Figure 1:**
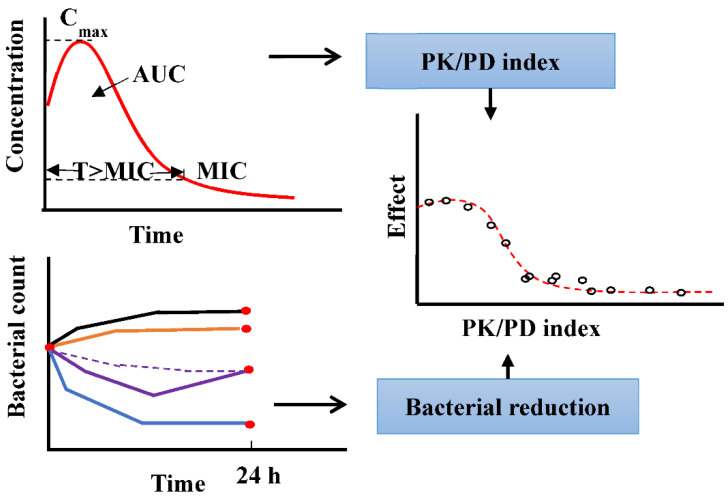
Method to derive the PK/PD index using the empirical PK/PD model.

The pharmacokinetic/pharmacodynamic (PK/PD) model is a recognized tool for determining the dosage regimen and guiding clinical medication. The PK/PD model of antimicrobial agents can describe the triangular relationship between the potency of a drug against a micro-organism, subject exposure to a drug, and drug effects [3] and contributes to the adjustment and optimization of dosage regimens, aiming to provide enough antibacterial effect for an infection caused by drug-resistant bacteria [4]. The PK/PD model of antimicrobials has been categorized by three PK/PD indices, which are comprised of a summary endpoint of drug exposure correlated with the minimum inhibitory concentration (MIC). The standardized notations for the PK/PD indices are AUC_0–24h_/MIC, C_max_/MIC, and T > MIC, where AUC_0–24h_/MIC is the ratio of the area under concentration related to the MIC within a 24 h period, C_max_/MIC is the maximum concentration related to the MIC, and T > MIC is the cumulative percentage of the 24 h that the concentration is above the MIC. Over recent decades, the PK/PD indices have been applied as a cornerstone of dose selection for antibiotics; however, this approach is associated with several drawbacks: 

(1) The limitation of the MIC. The MIC is determined at only one time point (over a 16–18 h period), with a low initial inoculum (i.e., usually in the absence of resistant populations), and utilizing a constant inoculum antimicrobial concentration, which is opposed to the dynamic changes in the in vivo condition [5]. The MIC-based method ignores the time-course of bacterial killing and is unable to predict the emergence of resistance. Additionally, with the two-fold dilution technique and visual inspection, measured and subjective errors may exist in the MIC results, inducing substantial uncertainty and variability. Thus, MIC is not an appropriate quantitative PD index to characterize concentration–effect relationships [6]. 

(2) PK/PD indices is established by correlating the reduction in bacterial count 24 h after an initial inoculum count [7]. It is an empirical method to select the best PK/PD index to predict the in vivo clinical efficacy and determine the optimal dosage regimen (as shown in Figure 1). The endpoints lack detailed information about the time course of the individual PK and PD processes, and the PK/PD indices are highly reliant on the MIC; thus, the drawbacks of the MIC (static concentration and uncertainty) are propagated in the indices. The PK/PD indices also vary between different patients, diseased conditions, and MIC uncertainty. For example, the most correlated PK/PD index of cefuroxime, a time-dependent antimicrobial, changes between T > MIC and AUC/MIC at different conditions [8]. Additionally, the dosage regimen, determined by the PK/PD index, cannot provide information about resistance. Thus, applying PK/PD indices to design the dosage regimen seems questionable [9].

Mathematical models are important decision-supporting tools in medicine and public health [7]. An in silico trial based on the PK/PD model can serve as primary evidence for the approval of a labelled dosage regimen for one patient. In addition, the US Food and Drug Administration (FDA), European Medicines Agency (EMA) and Centre for Drug Evaluation of China encourage the adaptation of mathematical models for drug research and development [10].

A semi-mechanistic PK/PD model was applied to alleviate the shortcomings of the empirical model. The semi-mechanistic PD model can parameterize the processes of bacterial growth and antibacterial effect and can be integrated with the PK model to simulate the growth, death, and resistance emergence of the bacterial population under different antibacterial exposures. Compared with the mechanistic PK/PD model, the semi-mechanistic PK/PD model focuses on the macroscopic description of the microbial system. As an example, for AMR, the mechanistic PD model is expert in describing the process of protein function, which can influence the antimicrobial effect, and the semi-mechanistic PK/PD model can simply the process and only describe the antimicrobial resistance phenomenon. A semi-mechanistic PK/PD model, where prior mechanistic understanding of the system is coupled with experimental data, may offer additional information in the model extrapolations compared to an empirical model, while requiring less prior knowledge [11]. The semi-mechanistic PK/PD model is devoted to improving our understanding of the emergence, development, and spread of AMR and is a valuable tool for optimizing the dosage regimen of antimicrobial agents.

In this review, we introduce several semi-mechanistic models and analyzed different factors that would influence the assessment of antibacterial effect, such as the inoculum effect and host response. Additionally, we summarize the application of various semi-mechanistic PK/PD models in alleviating the emergence of AMR.

## 2. Components of Semi-Mechanism PK/PD Model

Typically, a semi-mechanistic PK/PD model is composed of three basic components: (i) a component describing the PK of the drug, (ii) a component describing the bacterial kinetics, and (iii) a component describing the antibacterial effect. 

The PK model is applied to describe the adsorption, distribution, metabolism, and excretion progress in the body. The compartmental model represents the body as a system of one or more compartments, which does not correspond to the physiological and anatomical mechanism [12]. For the mechanistic model, the physiological-based PK (PBPK) model describes the processes of chemical absorption, distribution, metabolism, and excretion (ADME) based on physiological and biochemical mechanisms [13]. Population PK modelling aims to use the techniques of model development to describe the population of interest. The process of modelling seeks to identify covariates that may be associated with potential sources of variability, particularly between individuals of relevance [14]. The ability to extrapolate dose and simulate the concentration–time curves using the mechanistic model is a great advantage. 

In this review, we overview the components of bacterial growth and antibacterial effect, focusing on the AMR mechanisms. 

### 2.1. Bacterial Growth Model

The simplest model, which describes the bacterial kinetics by a first-order rate constant for bacterial growth (*k_growth_*) and a first-order rate constant for bacterial death (*k_death_*), is shown in the Equation (1) [15]. B is the number of bacteria count by agar determination. The first-order rate constant for observed growth (*k_net_*) is calculated as *k_net_ = k_growth_ – k_death_*, since it is difficult to separate micro-organisms for growth or death using the PD experiment.

(1) $$\frac{dB}{dt}=k_{net}\times B$$

A saturable non-linear model is also used to describe bacterial growth, as Equation (2) shown as Equation (2), where *VG_max_* is the bacterial maximum growth per time and *B*_50_ is *B* at which the bacterial growth is half-maximal [16].

(2) $$\frac{dB}{dt}=\left(\frac{VG_{max}}{B_{50}+B}\right)\times B$$

In the absence of an antimicrobial, bacteria grow exponentially until they reach a stationary bacterial level. It has always been thought that a limitation of nutrients leads to the stationary level. A logistic function can be used to describe capacity-limited growth curves, where *B_max_* is the maximum number of bacteria in the system, as Equation (3) shown [17]. However, the application of capacity-limited micro-organism growth to capture the time course of bacteria using the in vitro static and dynamic model indicated that nutrients are not the main reason [8].

(3) $$\frac{dB}{dt}=k_{net}\times \frac{B_{max}-B}{B_{max}}B$$

Another approach to model capacity-limited growth is the division of micro-organisms into two subpopulations, one being a growing subpopulation and the other being a resting subpopulation. Bacteria in the growing stage are transformed into the resting stage when the system is approaching stationary (Equation (4)). The transformation could be well described by a constant rate [18].

(4) $$\frac{dS}{dt}=k_{growth}\times S-k_{death}\times S-k_{SR}\times S+k_{RS}\times R\;\frac{dR}{dt}=k_{SR}\times S-k_{RS}\times R-k_{death}\times R$$

where *S* and *R* are the bacterial number for the growing subpopulation and resting subpopulation, respectively, and *k_SR_* is a constant parameter that simulates the transfer from the growing stage into the resting stage. 

### 2.2. Antibacterial Effect Model

**Figure 2:**
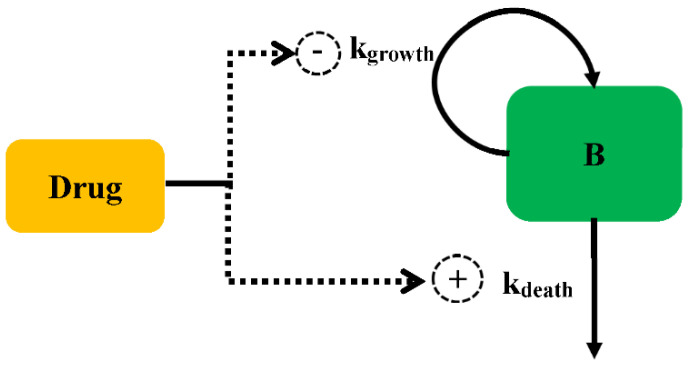
Schematic illustration of antibacterial effect against bacteria.

The antibacterial effect can be modelled for either increasing the death rate or decreasing the growth rate, as shown in Figure 2. The antibacterial effect can be assumed to be non-linearly dependent on the antimicrobial concentration and is modelled by a sigmoid *E_max_* model (Equation (5)):

(5) $$Effect=\frac{E_{max}\times C^{\gamma }}{EC_{50}^{\gamma }+C^{\gamma }}$$

where *E_max_* is the maximum effect on death rate, *EC*_50_ represents the concentration of drug that produces half of *E_max_*, *γ* is the coefficient, and *C* is the antibacterial concentration. 

However, if there is insufficient information to estimate *E_max_*, the following power equation can be used to describe the antibacterial effect according to Equation (6):

(6) $$Effect=Slope\times C^{\gamma }$$

Drug-resistant bacteria may occur during therapy and influence clinical perspective, with unrationed antibacterial use for many decades being the main reason leading to the selection for resistance [19]. The semi-mechanistic PK/PD model can describe the relationship between drug exposure and the emergence of resistance. In theory, by increasing *EC*_50_ or decreasing *E_max_*, the resistance can be captured in the PK/PD model. If high drug concentrations can suppress the resistance, the increase in *EC*_50_ is used in the model. By contrast, the decrease in *E_max_* is more suitable when the increasing dose cannot eliminate resistance [20].

#### 2.2.1. Persistent Resistance

Bacteria can exhibit phenotype tolerance, whereby they can survive under antibacterial treatment without acquiring new mutations. Generally, a proliferating susceptible subpopulation always has a small fraction of non-proliferating subpopulations (persisters). In the absence of antimicrobial drugs, persisters can switch to the proliferating state, which can cause post-treatment relapses and enable the development of genetic resistance. Unlike genetic resistance, persistent resistance is non-inheritable, although the frequency of persisters in a population can be genetically determined [21].

**Figure 3:**
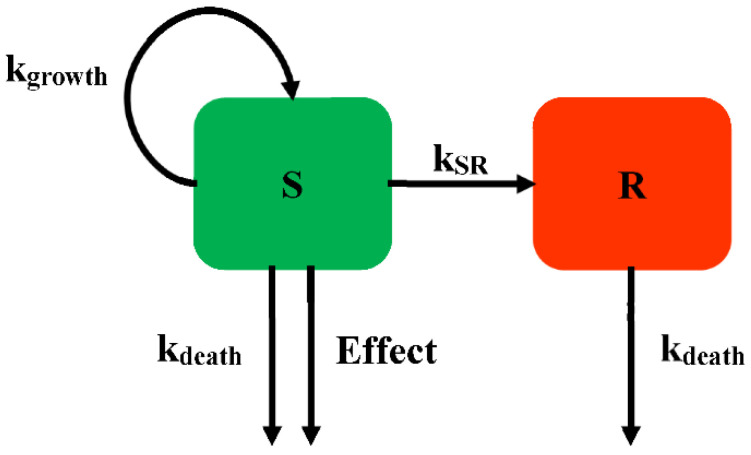
Schematic illustration of persistent resistance (Model 1).

The persisters were modelled according to the idea of a phenotypic switching between a normal growth subpopulation and a persistent subpopulation. Nilsen described two states of bacteria growing under antibiotics: one growing as a susceptible subpopulation and the other as a resting resistant subpopulation (Model 1, Figure 3) [18]. Different states are assumed to have the same death rate (*k_death_*). The antimicrobial drugs only influence the growing state. With a first-order constant (*k_SR_*), bacteria can transform from the growing state to the resting state. This model can explain the biphasic bacterial concentration-time curves, with a rapid killing rate followed by a decline in the killing rate over time. 

For Model 1, the susceptible subpopulation is inhibited by antimicrobials in the growing state. The resting state, which is unsusceptible to antibacterial, is employed for the model describing the biphasic kill, which can always be found in time-killing curves. Nielsen successfully described the time-course of *Streptococcus pyogenes* exposed to five different antimicrobial drugs. In addition, the fine-tuning of this model structure allows further study in the application of the semi-mechanistic model.

**Figure 4:**
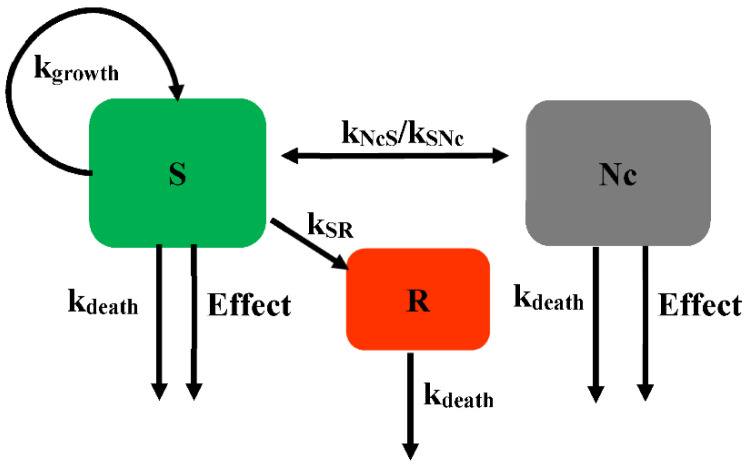
Schematic illustration of persistent resistance (Model 2).

Khan et al. further developed Model 2 (Figure 4) to describe the killing kinetics for *Escherichia coli* (*E. coli*) exposed to ciprofloxacin [22], with each subpopulation including three states for drug-susceptible growing bacteria (*S*), resting non-growing bacteria (*R*), and non-colony-forming drug-susceptible bacteria (*Nc*). The *Nc* subpopulation is rendered non-growing under the influence of the drug and is unable to grow on agar plates. This *Nc* state is most likely due to the formation of filaments, which is well known when *E. coli* is exposed to ciprofloxacin. The adaption of the *Nc* state has significantly improved the description of the time-killing experiment [22]. If there is insufficient drug to kill the filament bacteria, they may revert to susceptible growing bacteria at constant rates (*k_NcS_* or *k_SNc_*). The bacteria in the *S* and *Nc* states are both affected by the drug. The resting subpopulation is assumed to have a different *EC*_50_ compared with the susceptible subpopulation. The kinetics of the bacteria with time for different compartments is described in Figure 4. 

The mathematical equation describing the bacterial count for the different subpopulations of Model 2 is shown in Equations (7)–(9).

(7) $$\frac{dS}{dt}=k_{growth}\times S-k_{death}\times S-Effect\times S-k_{SR}\times S-k_{SNc}\times S+k_{NcS}\times Nc$$

(8) $$\frac{dR}{dt}=k_{SR}\times S-k_{death}\times R$$

(9) $$\frac{dNc}{dt}=k_{SNc}\times S-k_{NcS}\times Nc-k_{death}\times Nc-Effect\times Nc$$

where the *Nc* subpopulation is assumed to be non-growing under the influence of drug and unable to grow on agar plates. The transformation rates between the susceptible growing subpopulation and the *Nc* subpopulation are *k_SNc_* and *k_NcS_*.

**Figure 5:**
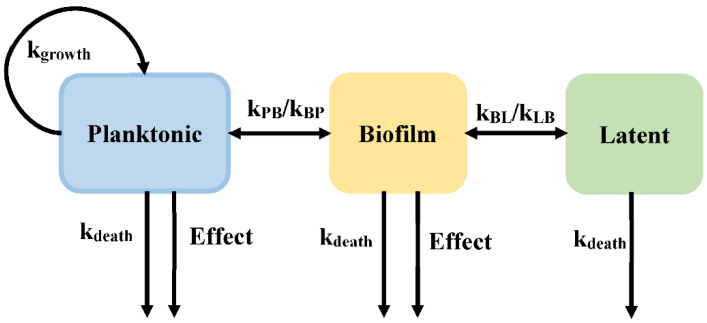
Schematic illustration of persistent resistance (Model 3).

Biofilms are sessile communities of bacterial cells, enclosed in an exopolymer matrix and adherent abiotic surfaces. In developed countries, 65% of all infections are caused by biofilms [23]. Biofilm plays an important role in immune evasion and tolerance toward antimicrobial agents, leading to persistent and chronic infections. Sou et al. proposed a model incorporating a semi-mechanistic description of biofilm development to evaluate drug action on bacteria (Model 3, Figure 5), with bacteria in the planktonic state (*P*), the biofilm state (*B*), and the latent state (*L*). The planktonic state represents the fast-growing bacteria. The planktonic state can transit into a biofilm state, in which the bacteria can readily transform into a non-growing latent state, which is tolerant to antibacterial. The planktonic and biofilm states are both susceptible to antibiotics [24]. 

Sou et al. assessed different equations that are suitable for use in the semi-mechanistic model describing the drug effect of *Pseudomonas aeruginosa* chronic lung infections. For example, different growth equations were outlined as exponential, capacity-limited, Gompertz, and logistic growth, as equation 9 shown. Using the Akachi information criterion (AIC) and goodness-of-fit between prediction and observation, the best equation (capacity-limited growth function) was selected to describe planktonic cell growth. In addition, they also evaluated the equation relating to the transport rate from the planktonic state to the biofilm state (*k_PB_*, as shown in the Equation (10)) and the antibacterial effect (*Effect*).

(10) $$\begin{array}{c} k_{PB}=k_{PB0}\times \frac{P+B+L}{B_{max}}\\ k_{PB}=k_{PB0}\times t\\ k_{PB}=k_{PB0}\times \left[1-\exp\left(-t\right)\right] \end{array}$$

where *P*, *B*, and *L* represent the bacterial count in the planktonic state, biofilm state, and latent state, respectively. The transition rate (*k_PB_*) is assumed to have different functions: a first-order transition dependent on the amount of bacterial in the system or increasing with time in a linear or a non-linear manner. 

**Figure 6:**
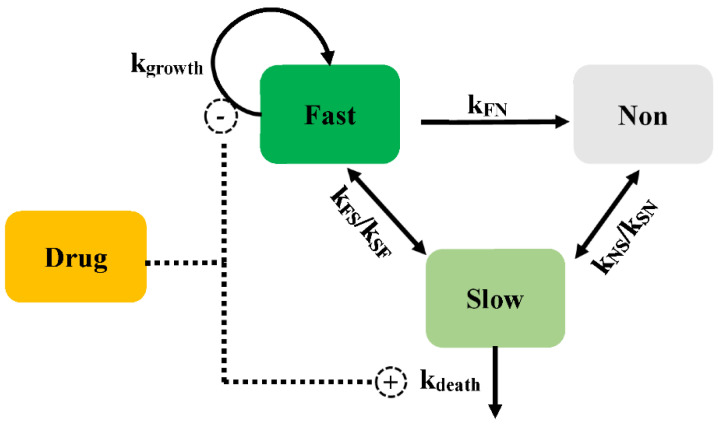
Schematic illustration of persistent resistance (Model 4).

*Multistate tuberculosis* (*M. tuberculosis*) exists in growing and dormant forms in a stationary-phase. In addition, both in vitro and in vivo, *M. tuberculosis* exists in a stage that is not able to form colonies on solid media, while being able to multiply in liquid media [25]. The PD model has been described as consisting of fast- (*F*), slow- (*S*), and non-multiplying (*N*) bacterial states (Model 4, Figure 6) [26]. The mathematical equation was according to Equations (11)–(13). Except for the transformation from fast- to slow-multiplying states, bacteria can interconvert into each state with a first-order rate. The transfer rate from fast- to slow-multiplying bacteria (*k_FS_*) is estimated using a linear or non-linear manner dependent on time. The time courses of the bacterial count change are described in the following equations. They separate the drug effect on the inhibition of the growth of fast-multiplying states and the simulation of the death of slow-multiplying states. The *E_max_* and *EC*_50_ are assumed to be different values for fast- and slow-multiplying states, and no death rate is described in the mechanistic PD model of *M. tuberculosis*.

(11) $$\frac{dF}{dt}=k_{growth}\times \left(1-Effect_{F}\right)\times F+k_{SF}\times S+k_{NF}\times N-k_{FS}\times F-k_{FN}\times F$$

(12) $$\frac{dS}{dt}=k_{FS}\times F+k_{NS}\times N-k_{SF}\times S-k_{SN}\times S-Effect_{S}\times S$$

(13) $$\frac{dN}{dt}=k_{FN}\times F+k_{SN}\times S-k_{NS}\times N-k_{NF}\times N$$

where *k_FS_*, *k_SF_*, *k_FN_*, *k_NF_*, *k_SN_*, and *k_NS_* are the transport rates among different subpopulations. F, S, and N are the bacterial numbers in the fast-, slow-, and non-multiplying subpopulations. *Effect_F_* and *Effect_s_* are the antibacterial effects on the fast-multiplying bacteria and slow-multiplying bacteria, respectively.

#### 2.2.2. Pre-Existing Resistance 

Considering the mutant rate and initial inoculum, the bacterial system more likely presents a pre-existing subpopulation for high initial inoculum [27]. A longitudinal model consists of a susceptible subpopulation and a pre-existing resistant subpopulation (Model 5). In each subpopulation, the bacteria exist in two states: proliferating antibiotic susceptible bacteria and resting non-proliferating bacteria. Both are assumed to have the same growth rate, death rate, and maximum killing rate (*E_max_*), but the two subpopulations differ in the values of *EC*_50_. The drug concentration that produces 50% of *E_max_* for the susceptible subpopulation is much smaller than for the resistant subpopulation. The bacterial count change for the susceptible and resistant subpopulations can be defined by the Equations (14) and (15). This model is also used to describe the dynamic kinetics of intestinal flora in vivo [28].

(14) $$\frac{dS}{dt}=k_{growth}\times S-\frac{E_{max}\times C^{\gamma }}{EC_{50,S}+C^{\gamma }}\times S-k_{death}\times S$$

(15) $$\frac{dR}{dt}=k_{growth}\times R-\frac{E_{max}\times C^{\gamma }}{EC_{50,R}+C^{\gamma }}\times R-k_{death}\times R$$

where *EC*_50,*s*_ and *EC*_50,*R*_ represent the concentration of drug that produces half of *E_max_* for the susceptible subpopulation and the resistant subpopulation, respectively.

**Figure 7:**
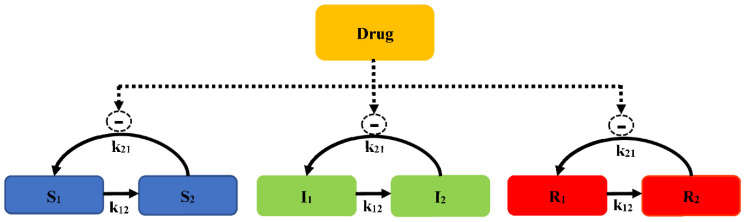
Schematic illustration of pre-existing resistance (Model 6).

There is a model that contains a susceptible subpopulation (*S*), an intermediate subpopulation (*I*), and a resistant subpopulation (*R*), which is also considered to evaluate the time-courses of bacteria counts (Model 6, Figure 7). 

A life cycle growth model is used to describe the underlying biology of bacterial replication. The bacterial replication for each subpopulation is defined as two states: preparing for replication (state 1) and immediately before the replication step (state 2). The replication rate constant (*k*_21_) is assumed to evaluate different susceptibility subpopulations. The total concentration of viable bacteria (*CFU_all_*) is calculated as Equation (16):

(16) $$CFU_{all}=S_{1}+S_{2}+I_{1}+I_{2}+R_{1}+R_{2}$$

The concentration of bacteria counts for the susceptibility subpopulation at state 1 is described as Equation (17):

(17) $$\frac{dS_{1}}{dt}=REP\times \left(1-Drug\right)\times k_{21}\times S_{2}-k_{12,S}\times S_{1}$$

where *k*_21_ is the replication rate constant, *k*_12_ is the growth rate constant, and REP is the replication factor, which is calculated as Equation (18):

(18) $$REP=2\times \left(1-\frac{CFU_{all}}{CFU_{all}+CFU_{max}}\right)$$

In the initial cultivation, REP represents a 100% probability of successful replication. As *CFU_all_* approaches the maximum bacterial size (*CFU_max_*), *REP* approaches a 50% successful replication, with the total viable count remaining constant. The antibacterial effect is described by the *E_max_* model. The concentration of bacteria counts for the susceptibility subpopulation at state 2 is described as Equation (19):

(19) $$\frac{dS_{2}}{dt}=-k_{21}\times S_{2}+k_{12,S}\times S_{1}$$

Similar equations are used for states 1 and 2 of the intermediate (I_1_ and I_2_) and resistant subpopulations (R_1_ and R_2_), but using different subpopulation parameters, such as *E_max_*, *EC*_50_, and *k*_12_. To better characterize *E. coli* when exposed to ertapenem (a broad-spectrum carbapenem), the model consists of the susceptible growing state (*S*), intermediate non-growing state (*I*), and resistant non-growing state (*R*) for two co-existing bacterial subpopulations (a susceptible subpopulation and a resistant population). Bacteria in *S* and *I* are drug susceptible, and bacteria can transform between states. This model well describes the results of an in vitro PD study of three *E. coli* strains, a native strain, an ESBL-producing strain, and an ESBL-producing strain with reduced expression of porins OmpF and OmpC.

#### 2.2.3. Adaptive Resistance 

Adaptive resistance is a specific class of non-mutational resistance that is characterized by its transient nature [29]. This resistant type is unstable and can be a repetitive and reversible occurrence, leading to a moderate broad-spectrum drug resistance. Adaptive resistance might explain the phenomenon of “baseline creep”, whereby the average MIC of a given medically important bacterial species increases steadily but inexorably over time. Tam et al. introduced an adaption factor (*α*) dependent on time and drug concentration by a saturable function of antimicrobial selective pressure, as shown in Equation (20) [30]:

(20) $$\alpha =1+\beta \left[1-e^{-e\left(t\right)\times t\times \tau }\right]$$

where *β* is the maximal adaption and τ is a rate constant of the adaption factor. An increase in adaptive factor can be implemented to result in a gradual increase in *EC*_50_, which can be considered as an evolution of the initial bacteria toward a resistant subpopulation. 

Jacobs et al. proposed a new adaption function based on an indirect model according to Equations (21) and (22) (Model 7) [5]. The time to adaptation is determined by the mean turnover time of adaptive resistance and is thus independent of drug concentrations. This adaptive function can reflect the situation that bacteria need to synthesize a protein to express a resistance mechanism, such as the expression of an efflux pump.

(21) $$\frac{d\left(Adaption\right)}{dt}=\left(\frac{S_{max}-C}{SC_{50}+C}-Adaption\right)\times k_{out}$$

(22) $$EC_{50}=EC_{50,base}\times \left(1+Adaption\right)$$

where *EC*_50,*base*_ is the antibacterial concentration causing 50% of *E_max_* in the absence of adaption, *S_max_* is the maximum fold-increase in *EC*_50_ due to adaptive resistance, *SC*_50_ is the concentration causing 50% of *S_max_*, and *k_out_* is the first-order rate constant for adaption. The adaption changes the extent of *EC*_50_ in response to the alteration of susceptibility for each subpopulation. 

**Figure 8:**
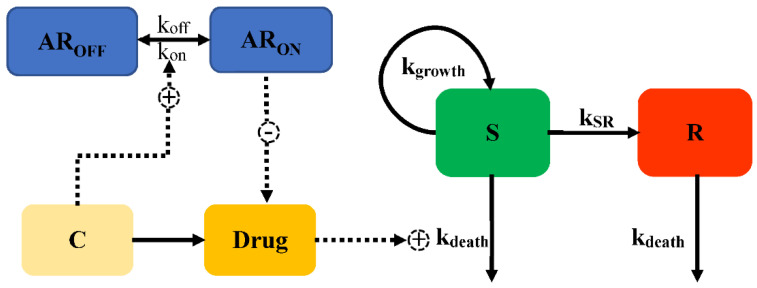
Schematic illustration of adaptive resistance (Model 8).

The adaptive resistance of aminoglycosides is a PD reversible process, which indicates that bacteria can revert to the initial state (susceptible to the antimicrobial drug) when drug exposure diminishes. Mechanistically, the expression of MexXY, an efflux pump related to the export of aminoglycosides, is turned down as drug exposure gradually diminishes, leading to susceptibility. Mohammad [31] introduced the development of adaptive resistance as a binding function, as Equations (23)–(25) shown, where the degree of binding resulted in a reduction of *E_max_* from the initial value (Model 8, Figure 8).

(23) $$\frac{dAR_{OFF}}{dt}=k_{off}\times AR_{ON}-k_{on}\times AR_{OFF}\times C$$

(24) $$\frac{dAR_{ON}}{dt}=k_{on}\times AR_{OFF}*C-k_{off}\times AR_{ON}$$

(25) $$E_{max}=E_{max,base}\times \left[1-\frac{AR_{on}}{AR_{ON}+AR_{50}}\right]$$

where *AR_ON_* describes the degree of adaptive resistance in the “on” state, *AR_OFF_* represents the adaptive resistance in the “off” state, *AR*_50_ is the value of *AR_ON_* when *E_max_* is reduced by 50%, and *k_on_* and *k_off_* are the constants of the reversal rate of adaptive resistance. Initially, *AR_ON_* is empty, and the whole fraction of a hypothetical amount is in *AR_OFF_*; upon drug exposure, the amount transfers into *AR_ON_*, with a rate constant *k_on_*. With a reduction of the antimicrobial, the bacteria gradually revert into the initial state (*AR_OFF_*) with *k_off_*.

## 3. Methods for the Development of Semi-Mechanism PK/PD Model

To develop an efficient semi-mechanistic PK/PD model, strain selection, in vitro time-killing experiments, and the establishment of a model need to be performed. Some details are listed below. 

Strain selection: One reference strain, and one susceptible and one less-susceptible clinical isolate are required. The selection of a reference strain can demonstrate reproducibility. It is better to choose virulent clinical isolates that can support translation to animals. Prior to establishing the model, the MIC needs to be determined, following the guidance, and the mutant frequency needs to be evaluated, which can assist with model establishment [27]. 

In vitro time-killing curves: Bacterial counts should be monitored repeatedly under multiple drug concentrations at different timepoints, including the susceptible subpopulation and the resistant subpopulation, in the in vitro time-killing experiment.

Establishing a model: Combined with the results of the in vitro experiment, a model, introduced as above, is selected. It is better to evaluate parameters and mathematical form, applying a non-linear mixed effects (NLME) model to run the semi-mechanistic PD model. Model performance is assessed by the evaluation of diagnostic plots, model fit, and the plausibility of estimated parameter values. In addition, the difference in objective function value (OFV) is employed: when the reduction in OFV is at least 10.84, the alteration is positive. AIC and Bayesian information criterion (BIC) can provide a reference to select an optimum model. In addition, some diagnostic graphs, such as goodness-of-fit and visual predictive curves (VPC), can assist in determining the model [32]. 

## 4. The Factors Affecting Model Establishment

In the PD experiment, some factors affect the establishment of the model, such as the inoculum effect, host response, and different types of PD data. Integrating with these factors, a dosage regimen can be derived comprehensively. This section discusses how the semi-mechanistic PD model integrates with these factors to capture the kinetics of bacterial count under drug exposure. 

### 4.1. Inoculum Effect

The inoculum effect is the phenomenon involving the attenuation of an antibacterial at a higher bacterial density of initial inoculum [17]. Numerous mechanisms have explained this PD phenomenon, which decreases the antibacterial effect: (1) due to the number of bacteria increasing, the concentration of antimicrobials is relatively decreased [17]; (2) during a high-density inoculum, a biofilm is established to hamper the antibacterial effect [18]; (3) in some cases, a high bacterial inoculum can mediate the expression of proteins that decrease antibiotic susceptibility, such as resistance enzymes or efflux pumps [19]; (4) higher concentrations of bacteria can increase the subpopulation of the pre-existing resistant subpopulation, while also enhancing the chances of a population spontaneously acquiring a mutation decreasing antibiotic susceptibility. A PK/PD model that can well capture this phenomenon would have an active effect on the selection of the dosage regimens.

Colistin is increasingly utilized to treat infections due to multidrug-resistance gram-negative pathogens, including *P. aeruginosa*, which greatly threatens human health. After displacing Mg^2+^ and Ca^2+^ from lipopolysaccharide binding sites, colistin disrupts the outer and cytoplasmic membranes. A model assessing the effect of Mg^2+^ and Ca^2+^ on the killing of *P. aeruginosa* by colistin was introduced by Bulitta et al. [33]. This model has also been successful in describing the inoculum effect. There are three different susceptibility subpopulations to colistin. To characterize the inoculum effect, all viable bacteria are assumed to synthesize and release freely diffusible signal molecules that can inhibit the killing effect. 

To evaluate the inoculum effect and the lag time of the killing, a mathematical model of Ceftazidime against *P. aeruginosa* was developed, involving two different subpopulations integrated with different growth states, as shown in Model 7. Compared with different PD models, a model that captures the life cycle of bacterial replication and autolysin activity can well describe the in vitro time-killing curves. Ceftazidime is assumed to bind with penicillin-binding proteins (PBPs) (*Stim_Drug,S_*), which stimulate the autolysin effect (*ALys_s_*), and the turnover of the autolysin effect causes the lag time of the killing. Autolysin activity is assumed to decrease the probability of successful replication, as shown in Equations (26) and (27):

(26) $$\frac{dALys_{s}}{dt}=\left[Stim_{Drug,s}-\left(1+\frac{S_{max,loss}\times C_{sig1}}{C_{50,Sig}+C_{sig1}}\right)\times ALys_{s}\right]\times k_{out}$$

(27) $$Stim_{Drug,S}=\frac{S_{max,S}\times C_{B}}{SC_{50}+C_{B}}$$

where *S_max,S_* is the maximum value of *ALys_s_* (when *S_max,S_* = 1, it indicates that the inoculum effect can completely inhibit replication at high drug concentrations), *SC*_50_ is the drug concentration at which the input of autolysin effect is half of the maximally stimulated, *S_max,loss_* describes the maximum extent of the inoculum effect at high signal molecule concentrations, and *C*_50,*sig*_ is the signal molecule that can perform half of the maximum extent of the inoculum effect.

### 4.2. Host Response

For Gram-negative bacteria, the release of inflammatory products, such as LPS/endotoxin, may increase due to the administration of antimicrobial drugs. This can be characterized as a simulator of the innate immune response. Beta-lactams bind with PBP-1 or PBP-3 (penicillin-binding proteins), which may lead to the release of a large amount of endotoxin upon final bacterial lysis. With the description of the mathematical method, Thorste et al. explored the endotoxin release in the context of drug administration or bacterial growth, by the quantitative link of two processes of bacterial growth [34]. Quantifying the release of endotoxin would assist in the design of a valuable dosage regimen. The bacterial growth model is described as Model 2, and the modelling of endotoxin release consists of (i) binary fission (growth [*G*]), (ii) antibiotic-induced killing (*K*), and (iii) natural bacterial death (*D*) as Equations (28)–(30) shown.

(28) $$\frac{dG}{dt}=k_{G,ETX}\times k_{growth}\times S-k_{el}\times G$$

(29) $$\frac{dK}{dt}=k_{K,ETX}\times \left(\frac{E_{max}\times C_{AB}^{\gamma }}{EC_{50}^{\gamma }+C_{AB}^{\gamma }}\right)\times S-k_{el}\times K$$

(30) $$\frac{dD}{dt}=k_{D,ETX}\times k_{death}\times \left(S+R\right)-k_{el}\times D$$

where *k_G,ETX_*, *k_K,ETX_*, and *k_D,ETX_* are assumed to be constant for different bacterial subpopulation release endotoxin, and *k_el_* represents the elimination rate, which is set to zero in the static model. For the in vitro dynamic model, a Gaussian function is employed to describe the transition from a susceptible state to a filamentous state (*k_filament_*) according to Equation (31).

(31) $$k_{filament}=e^{-\frac{{\left(C_{AB}-\theta_{MIC,F}\times MIC\right)}^{2}}{2}}$$

where *θ_MIC,F_* represents being centered around an antibiotic concentration given by the pathogen MIC times an estimated parameter.

(32) $$\frac{dF}{dt}=-\left(\frac{E_{max}\times C_{AB}^{\gamma }}{EC_{50}^{\gamma }+C_{AB}^{\gamma }}\right)\times F-k_{death}\times F+k_{filament}\times S+k_{growth}\times F$$

(33) $$\frac{dFK}{dt}=k_{F,ETX}\times \left(\frac{E_{max}\times C_{AB}^{\gamma }}{EC_{50}^{\gamma }+C_{AB}^{\gamma }}\right)\times F-k_{el,ETX}\times FK$$

where Equation (32) describes the formation of filaments. *k_growth_*, differentiated from the above, represents an increase in biomass, which leads to increased endotoxin release upon the antibiotic-induced killing of the filaments. Equation (33) described the antibiotic-induced killing of filaments.

It is difficult to directly describe the host response impact on enhancing the antibacterial effect or death rates of the bacteria. A biomarker is defined as a quantifiable biological parameter to guide diagnosis, the initiation, monitoring and cessation of therapy, and for prediction of clinical outcomes [35]. Different biomarkers were incorporated into the PK/PD model to quantify the immune system effect. Neutrophils and cytokines (TNF-α) [36] were selected as the biomarker to illustrate the effect of an immune cell on bacteria. An alternative method is to quantify the number of immune cells, with the host response assumed to be dependent on both the cell counts and bacterial counts, as shown in the Equation (34), which describes the time course of bacteria without drug exposure.

(34) $$\frac{dB}{dt}=k_{net}\times \frac{B_{max}-B}{B_{max}}\times B-\left(\frac{k_{ir}\times ANC}{ANC_{50}+ANC}\right)\times B$$

where *ANC* is the absolute neutrophil count, *ANC*_50_ is the ANC required to achieve 50% of the maximal kill rate, and *k_ir_* describes the maximum rate at which neutrophils can kill the pathogens.

The short- and long-term infection dynamics of TB are believed to be strongly influenced by the immune system response of an affected patient. A model designed with the mechanism of the immune system response to infection with *M. tuberculosis* was introduced by John et al. [37], which can be assumed in terms of the interaction between bacteria, immune cells in the alveoli of the lung, and the lymph nodes. 

Comprehending the mechanism and mathematically quantifying the time-course of the host, it is helpful to determine an optimal dosage for the patient [38]. 

### 4.3. The Types of Pharmacodynamic Data for the Model 

Initially, a semi-mechanistic PK/PD model was developed by the static time-killing curves, which only possess two variables: concentration and time. It is more suitably characterized by the mechanistic model than by MIC, with only one variable, concentration. Nielsen adapted Model 1 to successfully describe the kinetics of *Streptococcus pyogenes* exposure to five antibiotics [18]. However, due to the limitation of nutrients, the static time-killing curves are only represented within a 24 h period, and it lacks the ability to capture the resistance. To simulate in vivo conditions, where the drug is disposed and gradually changed, the dynamic model that can simulate the time-course of antibacterial concentrations observed in patients is used to establish the mechanistic PK/PD model. By adjusting pumps, the nutrients and toxic metabolites can be updated in the model, and a long-term culture can then be performed. The hollow fiber infectious model and the in vivo murine infection model were used to validated the predicted results by the semi-mechanistic PK/PD model, respectively, and revealed that the model can correctly predict the similar bacterial response (bacterial count change after 24 h exposure) in both in vitro and in vivo conditions [39].

It is a question of which types of data, static or dynamic time-killing curves, should be adapted to develop the semi-mechanistic PK/PD model. Comparing static or dynamic time-killing curves to support the precise parameter estimations of a PK/PD model, a study revealed that different settings between static and dynamic experiments did not significantly affect the growth kinetics of the bacteria [40]. Jacobs et al. distinguished six models with different mechanisms by static or dynamic time-killing curves. Dynamic conditions provided an accurate estimation for some parameters, and static conditions yielded more precise estimations. 

Furthermore, more studies employ static data to establish the mechanistic model and use dynamic data to validate the model [5]. To optimize the combined dosage regimens of meropenem-tobramycin against *P. aeruginosa*, a mechanistic model consisting of three different subpopulations is used to derive the dosage regimens [41]. Static time-killing curves are performed to characterize the effect of different meropenem and tobramycin doses, respectively, and an accurate hollow fiber infectious model is used to evaluate and validate the dosage regimen. According to the results, the in silico simulation of the antibacterial effect against *P. aeruginosa* and the suppression of resistance are similar to the results of the hollow fiber infectious model. 

## 5. Application of Model in Dosing Regimen, Combination Therapy, and Determination of Breakpoint 

With mathematical equations, the semi-mechanistic model can capture the time course of bacterial growth to evaluate the efficacy of the dose. The semi-mechanistic PK/PD model is widely applied for drug research and development. As shown in Table 1, we investigated the recent articles that adapt the semi-mechanistic PK/PD model. This model is an essential part of evaluating the efficacy of a preclinical drug and raising the rational of “old medicine”. Due to the complexity of drug resistance, more models employ various resistance mechanisms to describe the PD data. The semi-mechanistic PK/PD model was applied in the determination of dosage regimens, guidance of combination therapy, determination of the MIC-related breakpoint, and prediction of the kinetics of bacteria in the gut. This section introduces the application.

### 5.1. Dosage Regimen

Muhammad et al. adapted a whole-PBPK model combined with Model 2 to establish a PBPK/PD model of ciprofloxacin against *E. coli* [57]. This model can predict different time courses of bacterial killing in the extracellular fluid of different tissues. The results indicated the most antibacterial efficiency in the lung and kidney, which corresponds well to ciprofloxacin’s indications of pneumonia and urinary tract infections [57]. Sy et al. established a PK/PD model by the PBPK model and PD model (Model 2) to derive the best PD index (*f*T > C_T_) of avibactam against *P. aeruginosa* in combination with ceftazidime. Setting a threshold avibactam concentration of 1 mg/L, for the most ceftazidime-resistant strain, at least 500 mg q 8 h avibactam as a 2 h infusion can achieve at least a 2-log kill [58]. Kuepfer et al. described developing a PBPK/PD model for ciprofloxacin against *E. coli* in which the PD model allows the microbial growth to be quantified in the absence, as well as in the presence of ciprofloxacin-mediated inhibition. By comparing the PK profiles of concentrations in the lung and serum, 500 mg b.i.d. dosage regimen was already high enough to promote bacterial killing [59]. 

Lin et al. established a population PK model of a two-compartment model with linear elimination, assumed as plasma compartments and ELF (epithelial lining fluid) compartments. Model 5, established with the in vivo time-killing curves of the mouse lung infection model, was integrated with a population PK model to assess the efficacy of different dosage regimens of aerosolized colistin against *P. aeruginosa*. Deterministic simulations suggested that an inhalational dose of 60 mg colistin base activity every 12 h may be required to achieve ≥ 2 log 10 killings at 24 h after the commencement of inhaled therapy [54].

### 5.2. Combination Therapy

Semi-mechanistic PK/PD models that are developed based on in vitro single and combination experiments can be valuable for proposing a dosage regimen for drug combinations. Typically, antibiotic combinations are assessed to overcome or suppress the emergence of resistance and/or to increase efficacy. 

Zhao et al. [60] established a PK/PD model to quantify the interaction between polymyxin B (PMB) and minocycline (MIN) against multidrug-resistant *Klebsiella pneumoniae*. The description of the resistant state is an adapted Model 8. In addition, a basic additive interaction model was used in the selection of combination time-kill experiments of interest as Equation (35) described:

(35) $$k_{drug.COMB}=k_{drug.MIN}\times Inh_{AR.MIN}+k_{drug.PMB}\times Inh_{AR.PMB}$$

where *k_drug,COMB_* is the combined drug effect; *k_drug,MIN_* and *k_drug,PMB_* are the *k_drug_* of MIN and PMB, respectively; and *Inh_AR,MIN_* and *Inh_AR,PMB_* are the *Inh_AR_* of MIN and PMB, respectively.

Aranzana-Climent et al. [61] also employed Model 8 as the PD sub-model to describe the resistant mechanism. The drug interaction sub-model is described as Equation (36), as PD parameters are influenced by the presence of the other drug:

(36) $$EC_{50,MIN}=EC_{50,MIN\;alone}\times \left(1+\frac{INTP_{PMB}\times \left[PMB\right]}{\;EC_{50,INT-PMB}+\left[PMB\right]}\right)$$

where *EC*_50*,MIN*_ is the final *EC*_50_, which was influenced by polymyxin B; *EC*_50*,MIN alone*_ (mg/L) is the *EC*_50_ of minocycline, *INT_PMB_* (no unit) is an interaction factor, and *EC*_50*,INT-PMB*_ (mg/L) is the polymyxin B concentration needed to reach 50% of the interaction effect.

In addition, Brill et al. [62] overviewed the semi-mechanistic models to characterize the combined effect and outlined steps to establish models. Thirteen publications were reported about the combination mechanisms such as subpopulation synergy, different effect sites, and interaction functions. Subpopulation synergy was applied in several models, and independent kill rates of each drug were typically added using various types of interaction functions [62]. As the semi-mechanistic PK/PD model established, the evaluation of recommended dosing regimens and determination of an optimized dosage were performed. To expedite the application of semi-mechanistic PK/PD model, they uploaded the information for public availability of these models on DDMORE repository [63]. 

### 5.3. PK/PD Breakpoint and Cutoffs

The breakpoint or PK/PD cutoff can be determined by the semi-mechanistic PK/PD model. The MIC can be computed as a secondary parameter according to Equation (37).

(37) $$MIC=EC_{50}\times {\left(\frac{k_{growth}-0.29}{E_{max}-\left(k_{growth}-0.29\right)}\right)}^{\frac{1}{Gama}}$$

Pelligand et al. applied Model 1 to derive the PK/PD breakpoint of florfenicol against *Pasteurella multocida* and *Mannheimia haemolytica* with in vitro time-killing curves of different inoculum sizes. For both bacteria, the *f*AUC/MIC is selected as the PK/PD index in predicting bacterial killing with different MIC values. In silico simulation showed that average free plasma concentrations equal 1.2–1.4 times the respective MIC can perform with the maximum efficacy [55]. 

Iqbal determined MIC-developed breakpoints for the killing and suppression of resistance development in plasma and tissue sites, skin, and muscle. The clinical microdialysis data and in vitro time-killing curves of moxifloxacin against *S. aureus* and *E. coli* were used to establish the PK/PD model, and the different MIC-breakpoints of different antibacterial effects in different tissues were established for both bacterial species [50].

### 5.4. Prediction the Kinetic of Bacterial in Guts

The semi-mechanistic PK/PD model can present the dynamics of bacterial ecology and resistance in the gut intestine tract. The transmission dynamics of the resistance genes are commonly categorized into vertical vs. horizontal. In addition, many models were established to predict the susceptibility change of bacteria under antibiotic exposure in the gut. Erwin et al. used Model 5 to describe the growth of different bacterial subpopulations and investigated the susceptibility change of *E. coli* above and below the epidemiological cutoff in steers. They concluded that *E. coli* susceptibility is a strong indicator of how steers respond to antimicrobial drug treatment [28]. The semi-mechanistic PK/PD model can assist in predicting the number of resistant enterobacteria excreted. Nguyen et al. also used Model 5 to capture the complex relationships between dosage regimen, antibiotic fecal concentrations, loss of susceptible enterobacteria, and growth of resistant strains in the feces of piglets receiving different doses of ciprofloxacin for 5 days. For the clinically relevant dose of 15 mg/kg/day for 5 days, the total amount of resistant enterobacteria excreted was predicted to be reduced by 75% and 98% when reducing the treatment duration to 3 days and 1 day, respectively [64]. The modelling of the growth dynamics of multiple *E*. *coli* strains after ampicillin treatment have also been established by Ahamad et al., who revealed that the short period and dosing frequency do not influence the growth of resistant *E. coli* [65]. 

## 6. Overlook

The PK/PD model of antibacterial is an accepted method for optimizing the dosage regimen and provide some assistance in drug development and research. Accumulating studies focus on the semi-mechanistic model to design regimens and explore the resistant mechanisms. In addition, the semi-mechanistic PK/PD model can provide foresight in the pharmacological field, and a more rational and safer dose can be derived by the semi-mechanistic model for use in clinical phase I and II experiments. 

However, from a broader view in terms of future direction in this field, some thoughts need to be focused

1. Regulation. The official regulations need to be published, which will play the role of encouragement and guidance.
2. Education. It is very important to tell the modelers how to establish a model and judge the model. It is an efficient way to acquire the relevant knowledge from the tutorial. Rowland et al. summarized the inception, maturation, and future vision about Pharmacometrics and Systems Pharmacology. Twenty representative particles over the past 10 years were outlined [66]. Besides the tutorials, the software company and the public training courses also can offer some guidance. For example, many detailed courses can be found on the Metrum research group (www.metrumrg.com (accessed on 6 January 2022)).
3. Share. It is critical and necessary to publish the model code for the subsequent model applications. This will help modelers to learn the programming languages. Of course, the excellent forms of programming languages are also important. Mathematical models are widely used in various fields that would require more competent modelers.

In conclusion, we summarized the establishment of semi-mechanistic PK/PD models that can describe different antibacterial resistant mechanisms. The mathematical equation can describe the bacterial count change under drug exposure. With the development of computer technology, in silico simulation and prediction can extensively apply in the pharmacological field. Presumably, it offers another aspect to alleviate and avoid the emergence of drug resistance.

## Figures and Tables

**Table 1:** The semi-mechanistic PK/PD of antibacterial against different bacterial populations.

Antimicrobial Class	Drug	Bacteria	Resistant Species	References
Aminoglycosides	Gentamicin	*Staphylococcus aureus*	Adaptive resistance	[42]
		*Pseudomonas aeruginosa* *Acinetobacter baumannii*	Adaptive resistance	[43]
		*Escherichia coli*	Adaptive resistance Persistent resistance	[31]
		*Pseudomonas aeruginosa*	Pre-existing resistance	[44]
	Tobramycin	*Pseudomonas aeruginosa*	Persistent resistance	[24]
			Pre-existing resistance	[45]
Beta-lactams	Benzylpenicillin Cefuroxime	*Streptococcus pyogenes*	Persistent resistance	[18]
	Meropenem	*Pseudomonas aeruginosa*	Adaptive resistance	[30]
			Pre-existing resistance Persistent resistance	[46]
			Pre-existing resistance Persistent resistance Adaptive resistance	[47]
	Ertapenem	*Escherichia coli*	Pre-existing resistance Persistent resistance	[48]
	Ceftobiprole	*Staphylococcus aureus*	Persistent resistance	[49]
	Cefuroxime	*Escherichia coli*	Persistent resistance	[34]
Fluoroquinolones	Moxifloxacin	*Streptococcus pyogenes*	Persistent resistance	[18]
		*Staphylococcus aureus*	Pre-existing resistance Adaptive resistance	[50]
	Ciprofloxacin	*Staphylococcus aureus*	Pre-existing resistance	[51]
		*Pseudomonas aerugeinosa*	Adaptive resistance	[52]
		*Escherichia coli*	Pre-existing resistance Persistent resistance	[22]
	Enrofloxacin	*Escherichia coli*	Pre-existing resistance	[28]
Macrolides	Erythromycin	*Streptococcus pyogenes*	Persistent resistance	[18]
Polymyxin	Colistin	*Pseudomonas aeruginosa*	Adaptive resistance Persistent resistance	[53]
			Pre-existing resistance	[54]
Chloramphenicols	Florfenicol	*Pasteurella multocida* *Mannheimia haemolytica*	Persistent resistance	[55]
Tetracyclines	Eravacycline	*Escherichia coli* *Acinetobacter baumannii*	Adaptive resistance	[56]

## Data Availability

Not applicable.
